# Optimization of magnetic fluid hyperthermia protocols for the elimination of breast cancer cells MCF7 using Mn-Zn ferrite ferrofluid

**DOI:** 10.1007/s10856-023-06715-5

**Published:** 2023-03-14

**Authors:** Anand Bhardwaj, Kinnari Parekh, Neeraj Jain

**Affiliations:** 1 Dr. K C Patel R & D Centre, Charotar University of Science & Technology, CHARUSAT Campus, Changa- 388 421, Anand, India; 2 P D Patel Institute of Applied Sciences, Charotar University of Science & Technology CHARUSAT Campus, Changa- 388 421, Anand, India

## Abstract

**Abstract:**

The present study aimed to optimize magnetic fluid hyperthermia (MFH) protocols by standardizing MF incubation time, hyperthermic duration, magnetic field, and MFH sessions to achieve a better hyperthermic response for the profuse killing of human breast cancer cell cells MCF7. Magnetic nanoparticles and MF were characterized using XRD, VSM, and DLS. Induction heating was performed for 30 min at field strengths of 12.5 and 13.3 kA/m at a fixed frequency of 330 kHz with varying concentrations and incubation duration on MCF7 cells. Single and multiple sessions hyperthermia protocols were used to kill MCF7 cells and the cytotoxicity effect was analyzed using MTT assay. Single and multiple sessions MFH protocols were established to kill breast cancer cells utilizing 0.2 mg/mL MF at 13.3 kA/m field and 330 kHz frequency and maintaining the hyperthermic temperature of 43–45 °C for 30 min. The single session MFH revealed severe toxicity of MF leading to more than 75% of cell death after 24 h of MF incubation. Multiple sessions hyperthermia resulted in more than 90% killing of MCF7 cells after two consequent 3 h MF incubation with 3 h gap. Each 3 h of MF incubation was followed by 30 min of induction heating. Multiple sessions hyperthermia was effective in killing a larger cell population compared to the single session protocol. The results may help in optimizing protocols for the profuse killing of cancer cells of multiple origins, and aid in deciding futuristic in vivo MFH-based therapeutic strategies against breast cancer.

**Graphical Abstract:**

**Figure:**
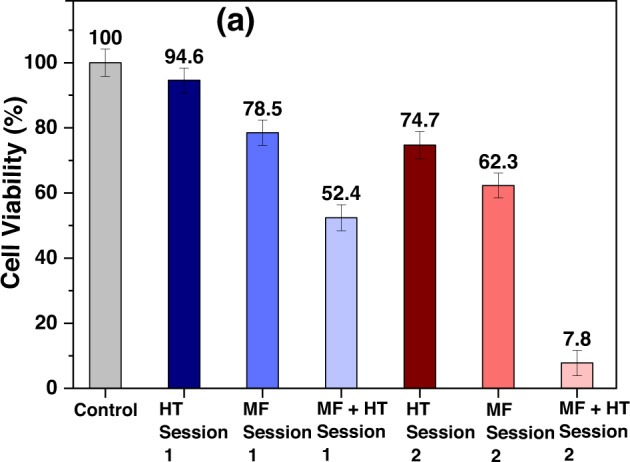
Variation in MCF7 cells’ viability due to HT, MF, and MF + HT in multiple sessions.

## Introduction

Cancer is a serious public health concern not only in terms of being the leading cause of premature death worldwide [1, 2] but also the challenges faced concerning its treatment modalities. As most cancer treatment options lead to minor-to-severe drug-induced toxicity, magnetic fluid hyperthermia (MFH) is emerging as an effective alternative therapeutic option due to its low-invasive nature and expected lesser side effects. Hyperthermia, in general, does serve as a complementary cancer therapy [3, 4]. However, only hyperthermia has limitations in controlling the temperature between 42–45 °C and also in preventing the overheating of the healthy tissue surrounding the tumor. In the case of MFH, this problem can be minimized as MFH-based therapy uses magnetic fluid (MF), a stable colloidal dispersion of magnetic nanoparticles in a non-magnetic liquid carrier medium such as water, under a high-frequency ac magnetic field to generate heat to kill cancer cells [5, 6]. A biocompatible magnetic fluid can be directly injected into the tumor and the heating response can be controlled by the particular choice of magnetic nanoparticles and with the external applicator parameters such as frequency and field strength. To date, clinical trials utilizing MFH have been conducted on recurring glioblastoma and prostate cancers only [7–11], and to treat various early-stage primary tumors, MFH protocols are required to be optimized for the effective killing of cancer cells while sparing nearby normal cells. Therefore, there is considerable scope for continual improvement of MFH-based treatment strategies both at the preclinical and clinical level with regard to the selection of magnetic particle composition, surfactant layer, controlled heating, concentration and time of ferrofluid treatment, magnetic field strength, the temperature distribution in the tumor, implantation methods, etc.

Further, along with glioblastoma and prostate cancer, breast cancer too is one of the most ferocious cancer that accounts for the largest number of cancer-associated morbidity worldwide [2]. Although significant progress has also been made in breast cancer treatment protocols over the years, markedly influenced by in vitro studies on the MCF7 cells [12], the development of drug resistance has been a major impediment to an effective breast cancer treatment. Hence, an MFH-based newer approach may serve as a boon to cancer patients.

The cytotoxic effects of MFH on MCF7 cells have been reported using a plethora of magnetic fluids [13–24]. Significant cytotoxicity of MCF7 cells was observed at higher concentrations of magnetic fluids; for example, 15 mg/mL [15] and 30–50 mg/mL [23] whereas some studies have used increased magnetic fields and frequency values to compensate for the low heating response at lower concentration of MNPs [14, 17, 21, 25]. On the other hand, high biocompatibility of chitosan functionalized LaF3:Yb,Er upconverting fluorescent nanotransducers in MCF7 cells has also been demonstrated [26]. Moreover, we have observed that breast cancer cells MCF7 show dissimilar responses and higher sensitivity to MFH compared to cervical cancer HeLa cells [20]. This suggests the need for an in-depth analysis of the hyperthermic effect of magnetic fluids to optimize MFH protocols for breast cancer cells.

Additionally, maintaining the hyperthermia window temperature of magnetic fluid is essential else temperature beyond 46 °C is known to be detrimental that leads to cell death through necrosis instead of apoptosis [27]. For this purpose, the manual intervention of the magnetic field applicator is required, however, this can be avoided by developing self-controlled heating of MNPs by limiting their Curie temperature. Thus, the heat dissipation from magnetic particles to its surrounding automatically stops when the temperature of magnetic particles reaches its Curie temperature. In this way, the magnetic particles act as a temperature control switch. Several promising temperature-sensitive magnetic fluids (TSMF) have been proposed in recent years including iron oxides doped with metals; for example, Fe_1-x_B_x_Fe_2_O_4_ (B = Mn, Co, Ni, Zn, Cu, Gd, Cr, etc.) [28–30], La_1−x_Sr_x_MnO_3_ [31], and Ni_1-x_-Cr_x_ alloys [32]. Out of these materials, Mn–Zn ferrite could be selected due to the ability to tune the Curie temperature by changing the Zn content, good magnetic response, and biocompatibility. Only a few studies have reported the effect of temperature-sensitive magnetic fluids on MCF7 cells. Thorat et al. [21, 25]. Using 1 mg/mL concentration of OA-PEG coated La_1-x_Sr_x_MnO_3_ (LSMO) MNPs [21], 55% cell death via MTT assay with 30 min MFH on MCF7 cells was observed at 23.9 kA/m magnetic field strength and 265 kHz frequency. Similarly, Qu et al. [25] used MnFe_2_O_4_ and Mn_0.6_Zn_0.4_Fe_2_O_4_ magnetic nanoclusters (MNCs) encapsulated in amphiphilic block copolymer of methoxy polyethylene glycol (mPEG) and polycaprolactone (PCL) and reported cell death up to 90% after 15 min of MFH on MCF7 cells using 0.2 mg/mL concentration of MNCs at 114 kHz frequency (magnetic field strength varied between 0 to 115 kA/m).

The above studies indicate the sensitivity of MCF7 cells toward MFH. However, no systematic study is reported that is carried out under the safety limit of H‧f with a minimum concentration of magnetic fluid. To the best of our knowledge, only one study has reported the effect of multiple sessions of MFH on MCF7 cells [23], where the observed cell death was less than 50%. Further, our preliminary observation suggests that breast cancer cells MCF7 show dissimilar responses and higher sensitivity to MFH compared to cervical cancer cell HeLa [20]. Hence arise the need for an in-depth analysis of the hyperthermic effect of magnetic fluids to optimize MFH protocols for breast cancer cells.

The present study attempts to address this research gap by using Mn_0.9_Zn_0.1_Fe_2_O_4_ nanoparticles based magnetic fluid (A91) on MCF7 cells and establishes the MFH protocols through a detailed study in terms of optimization of MF incubation time, hyperthermic duration, magnetic field, frequency and multiple sessions MFH. The therapeutic hyperthermic temperature (43–45 °C) was maintained for 30 min within the recommended safety limit of magnetic field and frequency (upper safety limit of H‧f = 5‧10^9^ A/m/s) as suggested by Hergt and Dutz [33].

## Materials and methods

The A91 magnetic fluid was synthesized using the chemical co-precipitation technique as described in our earlier study [20]. The crystallite size of particles obtained from the X-ray diffraction (XRD) pattern (data not shown) was 12.3 ± 0.5 nm. The magnetic response of the fluid was recorded using a vibrating sample magnetometer (VSM 7404, LakeShore, USA). The hydrodynamic size of the particles was measured using Dynamic Light Scattering having red LASER light of 633 nm wavelength (Zetasizer Nano-S90, Malvern, UK).

Induction heating (MFH) experiments were performed using EasyHeat LA-8310 (Ambrell, USA) operated at 330 kHz frequency inside a biosafety cabinet and utilizing a thermally insulated (using rubberized cork) 35 mm culture dish placed within the heating coil which contained the MCF7 cells and A91 fluid. The ambient temperature of the coil was maintained using water circulating chiller (Werner Finley, India) throughout the experiments. The temperature rise in a sample was measured via an optical fiber sensor attached to the instrument with one end dipping in the sample. The initial temperature of 29–30 °C was fixed for all the samples before recording the temperature data as a function of induction heating time. The medium-sized 2 × 2 turns Helmholtz coil with an inner diameter of 60 mm was used for the cell culture dish for which the magnetic field varied from 1.7 to 15.3 kA/m.

The breast cancer cell line MCF7 was procured from the National Center for Cell Science, Pune, India. The cells were grown in a 37 °C incubator (ThermoFisher, USA) with 5% CO_2_ and 95% relative humidity, using Minimum Essential Medium Eagle (MEM) (HiMedia, India) containing Earle’s salts, 2 mM L-Glutamine, 1 mM Sodium pyruvate, non-essential amino acids (NEAA) and 1.5 g/L Sodium bicarbonate, 10% heat-inactivated fetal bovine serum, 100 U/mL Penicillin, 100 g/mL Streptomycin, and 0.25 g/mL Amphotericin B (ThermoFisher, USA).

3-(4, 5-dimethylthiazol-2-yl)-2, 5-diphenyltetrazolium bromide salt (MTT) (Merck, USA) based cell viability assay on MCF7 cells was performed to calculate the 50% inhibitory concentration (IC_50_) as per the protocol routinely followed in our laboratory [20]. Briefly, 10^4^ cells/well were seeded in triplicates in a 96-well tissue culture plate and incubated for 24 h before being treated with magnetic fluid diluted in cell culture media at concentrations ranging from 0.5 mg/mL to 0.3 mg/mL. A set of three wells containing cells without MNPs served as untreated controls. After 24 h of incubation with magnetic fluid, the cells were rinsed with PBS to remove the suspended/free MNPs from the wells. After that, each well was filled with 300 μl of medium and 25 μl of MTT solution (5 mg/mL in PBS) followed by incubation for 3 h. After that, the media from each cell was withdrawn, and 100 μL dimethyl sulfoxide (Merck, USA) was added to each well to dissolve the crystal formazan that had formed. On an ELISA plate reader, the absorbance was measured at 570 nm (Molecular Devices, USA). The viability of the cells was estimated using Eq. 1.

1 $$\begin{array}{l}Cell\,viability\,{{{\mathrm{\% }}}} = \\ \frac{{average\,absorbance\,from\,treated\,cells\,after\,MF\,treatment\,in\,triplicates}}{{average\,absorbance\,from\,control\,cells\,in\,triplicates}} \times 100\end{array}$$

### Protocol for Single Session Hyperthermia on MCF7

In the single session hyperthermia, MCF7 cells grown on culture dishes (CDs) were treated for 1, 3, 6, 12, 18, and 24 h with A91 magnetic fluid at the concentration of 0.2 mg/mL and later placed under a 13.3 kA/m magnetic field at 330 kHz frequency to achieve the hyperthermic window of 43–45 °C which was maintained for 30 min.

**Fig. 1:**
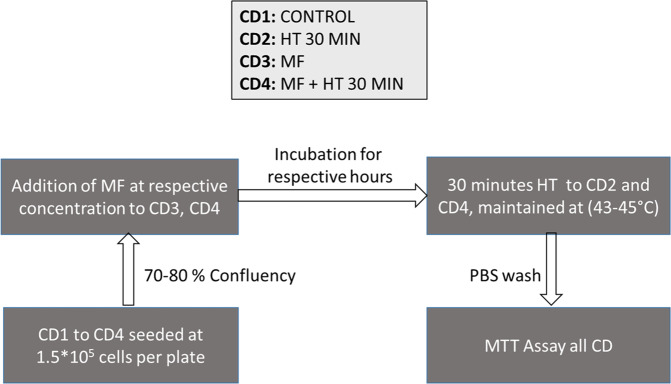
Flowchart of single session hyperthermia experiment, where CD Culture Dish, HT Hyperthermia, MF Magnetic Fluid

Briefly, 0.15 million cells were seeded in four culture dishes (CDs) numbered as (1) control cells without MF and without hyperthermia (HT); (2) cells without MF, with 30 min HT; (3) cells with MF, without HT; and (4) cells with MF and a 30 min HT. Once the MCF7 cells reached an approximate confluence of 80%, MF was added to the relevant CDs and were allowed to grow for their respective MF incubation duration. Induction heating was then performed on CDs 2 and 4. The hyperthermic window of 43–45 °C was maintained for 30 min. Following induction heating, the CDs were subjected to MTT assay for determining their viability. Each set’s results were compared to CD1 cells that served as untreated control. Figure 1 represents the flowchart of the single session hyperthermia protocol.

### Protocol for multiple sessions hyperthermia on MCF7

**Fig. 2:**
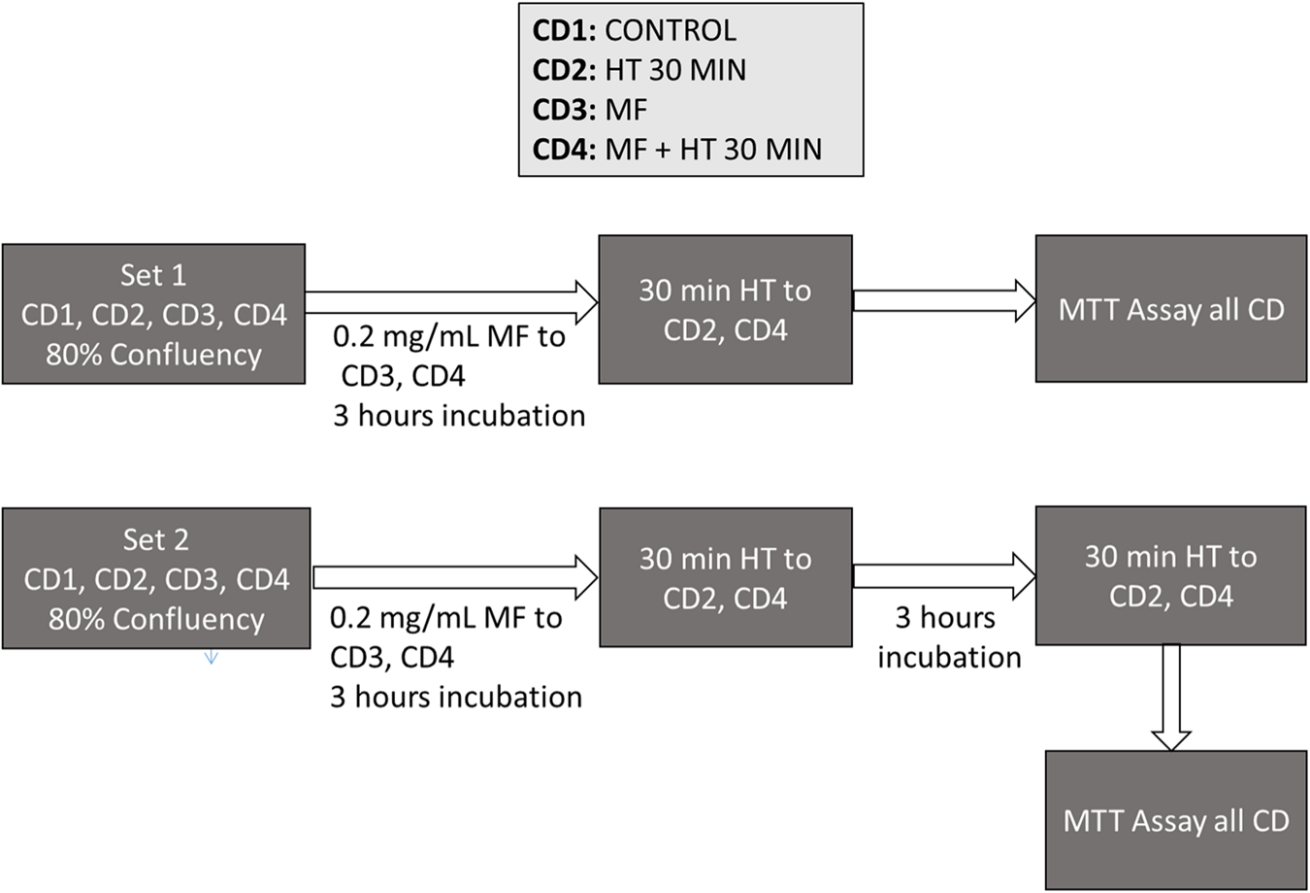
Flowchart of multiple sessions hyperthermia experiment, where CD Culture Dish, HT Hyperthermia, MF Magnetic Fluid

To study the effect of multiple sessions hyperthermia on MCF7 cells the concentration of magnetic fluid and HT duration were fixed at 0.2 mg/mL and 30 min, respectively. The experimental design consisted of two sets of culture dishes with four culture dishes in each set. The culture dishes were numbered as (1) control cells without MF and HT, (2) cells without MF but with HT, (3) cells with MF without HT, and (4) cells with MF and HT. The culture dish 1 in each set served as respective control. Briefly, when the cells reached an approximately 80% confluence after seeding 0.15 million MCF7 cells, MF was added to dishes 3 and 4 and incubated for 3 h along with the rest of the dishes. After 3 h, cells in dishes 2 and 4 of both sets underwent the first HT session of 30 min duration. After the HT session, all the cells of the first set of culture dishes were PBS washed and cell viability was assessed using MTT assay. The cells of the second set continued to grow for the next 3 h. Afterward, the cells of dishes 2 and 4 of the second set underwent a second HT session of 30 min. Thereafter, the cells of the second set were assayed for cell viability as per the procedure followed for the first set of culture dishes. Overall, the cells of culture dishes 2 and 4 of the first and second set received single- and double-time hyperthermia treatment of 30 min, respectively. The cell viability was determined using MTT assay after comparing treated cells against the control cells that were neither exposed to MF nor HT treatment. The results of each set were compared against the untreated cells of culture dish 1 of each set. Figure 2 represents a flowchart of steps involved in multiple sessions hyperthermia.

All the experiments were performed in triplicates. After calculating mean and standard deviations, the data were presented as mean values with standard error. The levels of significance were calculated using the Students’ *t*-test, and the results were considered significant if the *p*-value was less than 0.05.

## Results and discussion

### Magnetic fluid characterization

**Fig. 3:**
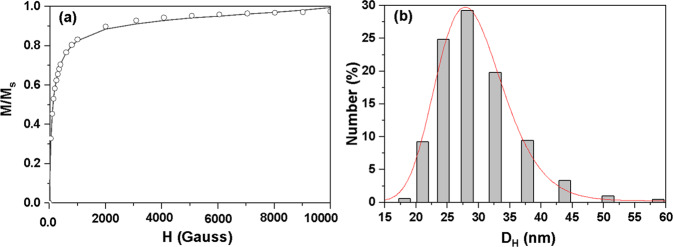
**a** Normalized magnetic response versus magnetic field for A91 magnetic fluid and **b** number distribution of particles versus hydrodynamic diameter of fluid

Figure 3a shows the normalized magnetic response as a function of the magnetic field for the parent fluid measured at room temperature. The magnetic parameters like domain magnetization, M_d_, magnetic particle size, D_m_, size distribution, σ, and saturation magnetization, M_s_ were calculated using Langevin’s fitting [34] of the obtained data. The best-fitted values were obtained with D_m_ as 10 nm, M_s_ as 4 kA/m, M_d_ as 350 kA/m, and σ as 0.62. The absence of remanence, coercivity, and hysteresis, showed a typical characteristic of nanoparticles that were superparamagnetic at room temperature. The typical number distribution of magnetic fluid dispersed in Milli-Q water was obtained using dynamic light scattering (DLS) and shown in Fig. 3b. The number-weighted hydrodynamic diameter distribution obeyed the log-normal distribution function (line in Fig. 3b). The results showed polydispersed spherical particles with an average diameter of 28.9 ± 0.1 nm and the width of the distribution curve (σ) was found to be 0.19. The higher value of hydrodynamic diameter as compared to the crystallite size obtained from XRD could be due to the multiple layers of coating of surfactant as well as the possibility of formation of stable small aggregates upon dilution [35].

### Magnetic fluid hyperthermia parameter optimizations for MCF7 cells

In our previous study, we observed severe toxicity of A91 magnetic fluid at 0.35 mg/mL concentration and without hyperthermia on MCF7 cells killing almost 90% of them whereas only 47% of HeLa cells died under similar conditions [20]. It became apparent that the MF concentration used on HeLa cells could not be applied to MCF7 cells which could be due to the differences in the cell membrane properties, thereby affecting the uptake propensities of MF by two cell types. Besides, MF concentration, magnetic field strength, and hyperthermic duration also play important roles and require optimization to obtain maximum killing of MCF7 cells within the hyperthermic windows of 43 to 45 °C.

**Fig. 4:**
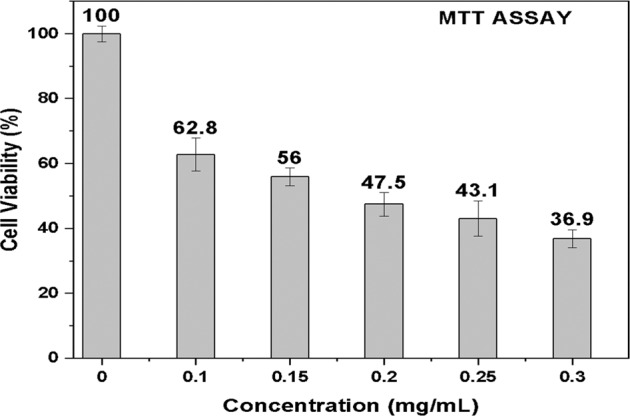
Cell viability as a function of concentrations of A91 fluid

Therefore to optimize the concentration, the cytotoxicity of A91 MF was examined on MCF7 cells in the range of 0.1, 0.15, 0.2, 0.25, and 0.3 mg/mL using MTT assay. The variations in the viability of MCF7 cells after 24 h of A91 treatment are shown in Fig. 4. Based on the results, the IC_50_ (half-maximal inhibitory concentration required to suppress viable cell counts in vitro by 50%) value was calculated. Upon increasing the concentration, cell viability decreases, and cell death increases. After fitting Hill’s equation to the dose-response curve [36], the MTT assay revealed IC_50_ values of 0.257 mg/mL. The IC_50_ on MCF7 cells obtained in the current study corroborates with the findings of Yang et al. [22], who used oleic acid and phosphorylated methoxy PEG-coated iron oxide MNPs on MCF7 cells and observed 35% cell death at 0.2 mg/mL concentration. Similar concentration-dependent cytotoxicity was observed by Lotfi et al. [16], who used chitosan-coated core-shell Fe_3_O_4_ MNPs on MCF7 cells and observed a cell death of up to 20% at 0.1 mg/mL concentration.

**Fig. 5:**
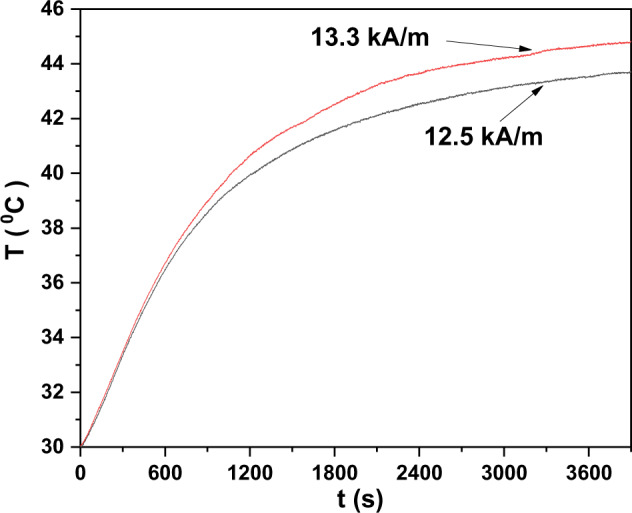
Temperature versus time response of 0.2 mg/mL A91 fluid at two different field strengths and a fixed frequency of 330 kHz

We observed a 52.5% cell death at 0.2 mg/mL concentration of A91. This concentration was selected for further study and hyperthermia was performed to find out if the temperature window of 43–45 °C could be reached and maintained by varying the magnetic field strength. Figure 5 shows the temperature response of A91 fluid diluted in the complete cell culture media (MEM) at 0.2 mg/mL concentration at two different field strengths of 12.5 and 13.3 kA/m at a fixed MFH applicator frequency of 330 kHz using 2 × 2 turn Helmholtz coil. It was observed that at a field of 12.5 kA/m, 43 °C was achieved in a long time of 48 min. Based on our previous experience where increased hyperthermia duration related to decreased MCF7 viability [20], we did not proceed with 12.5 kA/m magnetic field strength for further MFH study. A slightly higher magnetic field of 13.3 kA/m was set and the desired temperature of 43 °C was achieved in approximately 32 min that was maintained between 43 and 45 °C for the next 30 min. Subsequently, an MF concentration of 0.2 mg/mL, magnetic field strength of 13.3 kA/m, and hyperthermic duration of 30 min were selected for the single- and multiple sessions MFH study. The product of the magnetic field (13.3 kA/m) and frequency (330 kHz) obtained was 4.39*10^9 ^A/m‧s, a value well within the safety limit of 5*10^9^ A/m‧s for magnetic therapy.

### Single session hyperthermia - effect of incubation time on cell viability

**Fig. 6:**
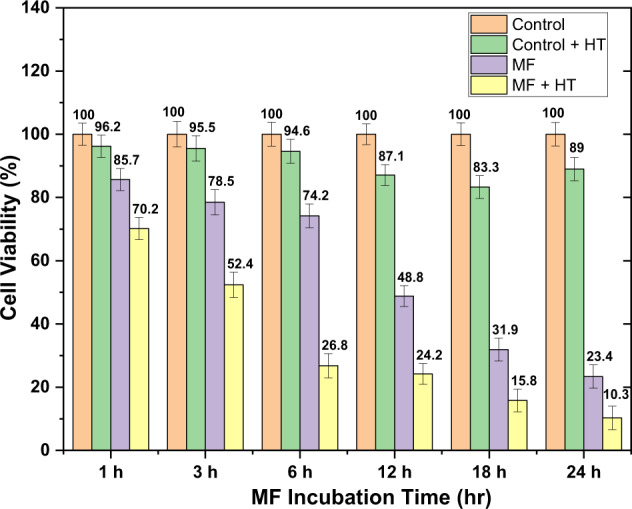
Variation in MCF7 cells’ viability due to HT, MF, and MF + HT. In the case of MF and MF + HT, the cells were treated with 0.2 mg/mL A91 MF and incubated at different time intervals (1, 3, 6, 12, 18, and 24 h). MFH was performed at 13.3 kA/m and 330 kHz for 30 min between 43 and 45 °C temperatures

The cytotoxic effect of HT, MF, and MF plus single-session HT on MCF7 cells is depicted in Fig. 6. An approximate 4 to 16% MCF7 cell death due to 30 min HT alone without MF was observed. Further, incubation of cells with magnetic fluid only, for 1, 3, 6, 12, 18, and 24 h resulted in 14.3, 21.5, 25.8, 51.2, 68.1, and 76.6% cell death, respectively. While with MF and 30 min HT, the cell death obtained was 29.8, 47.6, 73.2, 75.8, 84.2, and 89.7% after 1, 3, 6, 12, 18, and 24 h, respectively. Cell death increased with increasing MF incubation time as well as subsequent hyperthermia sessions. Maximal 90% cell death was achieved after 24 h of MF incubation time followed by MFH.

**Fig. 7:**
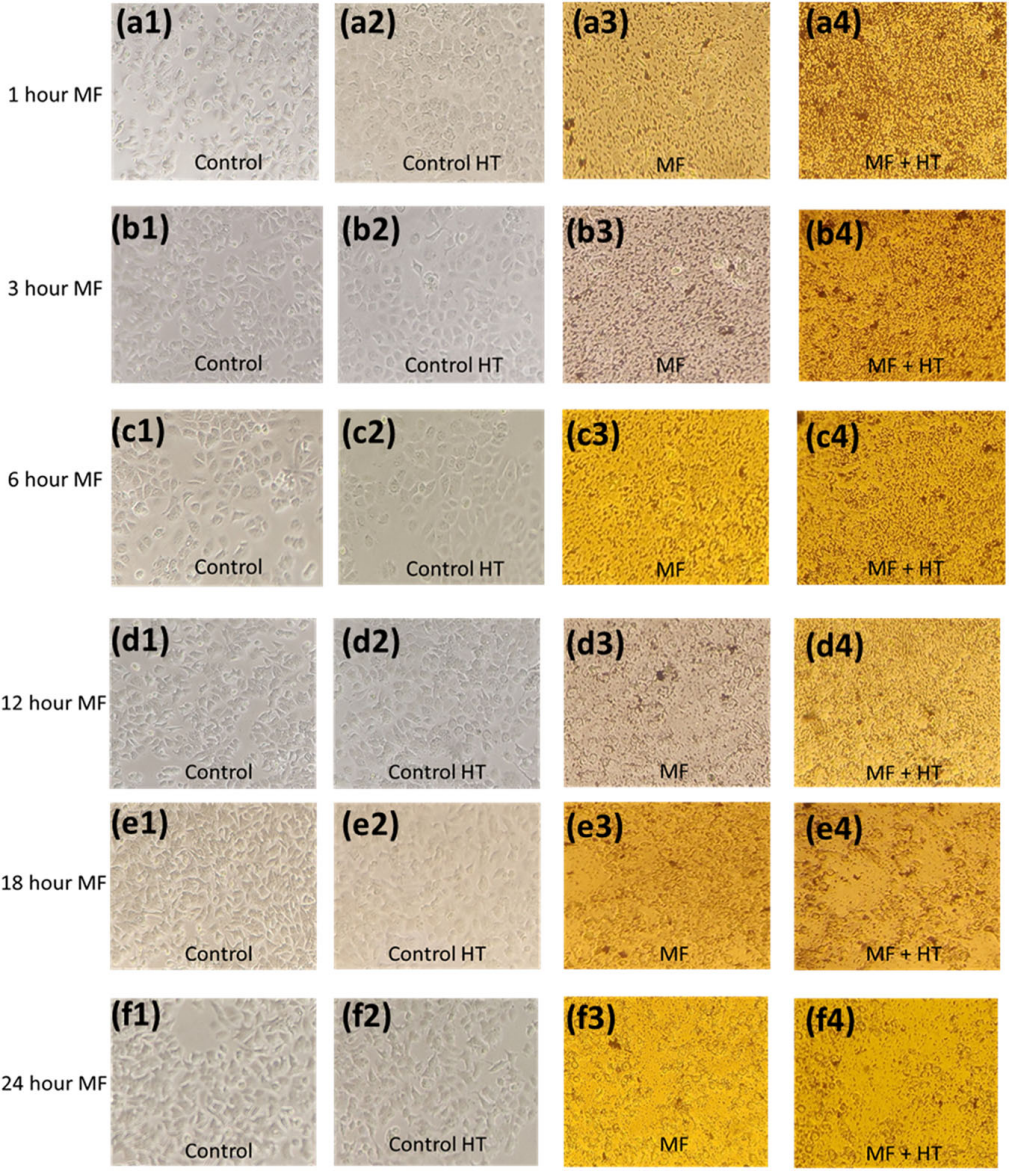
Microscopic image of MCF7 cells. **a1**–**f1** are images for untreated control cells. **a2**–**f2** are images for HT-only treated cells. **a3**–**f3** represents cells treated with MF for different time intervals. **a4**–**f4** represents cells treated with MF for different time intervals and 30 min of induction heating

The MTT assay-based cell death observations corroborated well with the visual examinations also. Figure 7 shows the images of control cells and cells that underwent HT, MF, and MF + HT treatment. After induction heating alone (Fig. 7 a2 to f2), the cells had almost similar morphology and number as the controls cells (Fig. 7 a1 to f1). However, a significant deterioration in cell morphology and reduction in cell count became evident in the MF and with MF + HT treated group as represented in Figs. 7 a3 to f3 and 7 a4 to f4, respectively. Cell death from approximately 80 to almost 100% by performing single session hyperthermia on MCF7 after 15 min to 48 h of MF treatment has been previously reported [13, 14, 21]. These groups either utilized iron oxide or manganese oxide-based MNPs, however, the product of H‧f reached was beyond the upper safety limit of 5‧10^9 ^A/m-s as suggested by Hergt and Dutz [33], whereas in our experimental set-up the product of H‧f was 4.39*10^9 ^A/m-s.

**Fig. 8:**
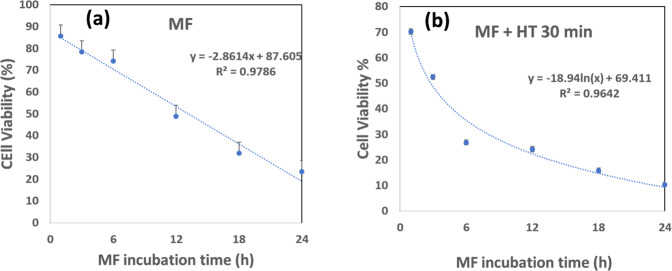
Variation of cell viability versus MF incubation time (**a**) cells treated with MF and (**b**) cells treated with MF + 30 min of HT. The dotted line shows log fit in the first case while linear fit in the second case

The graphical presentation of variation in cell viability with different MF incubation duration and MF + 30 min HT with logarithmic fitting is shown in Fig. 8a, b, respectively. The cytotoxic effect of MF is fitted with a linear equation with time (Fig. 8a) while the effect of induction heating revealed logarithmically fit (Fig. 8b). This signifies that MF alone can be highly toxic to the MCF7 cells over a period of time, irrespective of the concentration. However, systematic cell death with magnetic fluid hyperthermia was noticeable with increasing incubation time revealing almost 90% cell death after 24 h.

### Multiple sessions hyperthermia

Considering the profound cytotoxic effect of MF alone in the single session hyperthermia experiment, killing almost 77% of cells even without HT (Fig. 6), we designed multiple sessions HT protocol to obtain cell death following MF incubation together with induction heating. Furthermore, we observed that during a single experiment, a 3 h MF + HT treatment lead to around 50% cell death, so we hypothesized that a second HT session might lead to significant cell death. Therefore, we proceeded with a second session HT following 3 h of the first session as per the multiple session protocol described in the Methods section (refer Fig. 2). To our expectations, the second MF + HT session led to almost 92% cell death, which was around 20% higher compared to 6 h of MF incubation and single session MFH. Additionally, we also observed that cell death increased from <5% to approximately 25% from first HT only treatment to second HT alone treatment after the gap of 3 h. This further strengthens the sensitivity of MCF7 cells against high temperature, although within the proposed hyperthermic window of 43 to 45 °C, which should be contemplated while designing future MFH-based treatment protocols at in vivo level.

**Fig. 9:**
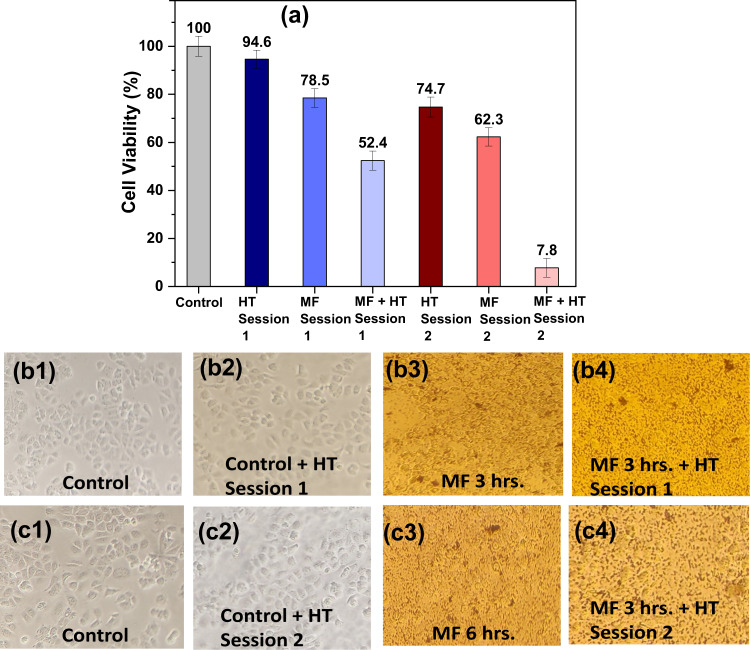
**a** Variation in MCF7 cells’ viability due to HT, MF, and MF + HT in multiple sessions. Microscopic image of cells in various conditions: (**b1**, **c1**) are control cells, (**b2**, **c2**) control cells treated with HT session 1 and 2, respectively, (**b3**, **c3**) control cells treated with MF for 3 h and 6 h., respectively while (**b4**, **c4**) are the cells treated with MF for 3 h then HT session 1 of 30 min duration was given, then the cells were incubated for another 3 h followed by HT session 2 of 30 min duration

Figure 9a shows MCF7 cells’ viability after multiple sessions hyperthermia performed at 13.3 kA/m, and 330 kHz, using 0.2 mg/mL A91 MF. Morphology of MCF7 cells is shown in Fig. 9 (b1 to b4) and (c1 to c4) after the first and second session HT, respectively.

The concentration of MF was fixed as 0.2 mg/mL A91 MF, and MFH was performed at 13.3 kA/m and 330 kHz for 30 min duration once the temperatures reaches 43 °C. In the first and second session, Fig. 9b1, c1 are control cells, whereas Fig. 9b2 is the image of the control cells after HT session 1 of 30 min duration and Fig. 9c2 represents cells’ image after HT session 2. It is seen that the image of cells is almost the same even after HT session. This indicates that HT does not affect the cells’ morphology when not treated with MF. The cells were incubated with MF for 3 (Fig. 9b3) and 6 h (Fig. 9c3), respectively. The morphology of cells is not clearly visible due to the background color of MF. HT was performed for 30 min in session 1 on cells treated with 3 h MF (Fig. 9b4). The second session of HT following 3 h of the first MF + HT was performed after 3 h incubation period. Fig. 9c4 shows the microscopic image of MF + HT 2. The number of cells reduced after every HT session confirming the killing of cancer cells due MFH.

The effect of multiple sessions of MFH on MCF7 cells was solely reported by Zhao et al. [23] using amino silane coated iron oxide MNPs at 40 mg/mL concentration on MCF7 cells and observed 17, 22, and 30% cell death after the first, second and third HT session, respectively, of 20 min each with 24 h of incubation with MNPs. The MFH was performed at 8.8 kA/m and 300 kHz with temperature-controlled at 43 °C for 20 min. In their study, though, no significant cell death was observed due to MF alone for up to 72 h, which may be ascribed to greater biocompatibility of amino silane coating compared to the lauric acid coating used in our study, the cell death due to HT reached up to 30% only. Other than breast cancer cells, the effects of multiple sessions MFH has been reported on human osteosarcoma cell line by Makridis et al. [37]. They used manganese ferrite MNPs on osteosarcoma cells Saos-2 at 0.25 and 0.5 mg/mL concentrations of MF with the hyperthermic duration of 6 min that reached 41–45 °C temperature in 4–6 min with a time interval of 48 h between the two HT sessions. This resulted in approximately 30 and 90% cell death due to the first MFH session (24 h of MF incubation) and second MFH session (72 h MF incubation), respectively.

Compared to both of these studies we have observed a significant cell death due to MFH at a lesser MF concentration and incubation time. No report to date is available on the effect of Mn-Zn ferrite temperature-sensitive MF on breast cancer cells MCF7. The study results suggest that two sessions of shorter duration HT using Mn-Zn ferrite-based MF may serve as a potent anti-cancer strategy, especially against breast cancer that needs to be further investigated at in vivo level. Although, cell death occurring due to HT between 43 and 45 °C is known to occur through apoptosis, further analyses to confirm the same after multiple sessions of HT using Mn-Zn ferrite MF are warranted.

## Conclusion

Present work reports the investigation and optimization of magnetic field strength, MF incubation time, and the number of HT sessions using temperature-sensitive Mn-Zn ferrites-based magnetic fluid to annihilate human breast cancer MCF7 cells. Single and multiple sessions MFH protocols were established using 0.2 mg/mL MF at 13.3 kA/m field and 330 kHz frequency for 30 min maintaining the hyperthermic temperature of 43–45 °C. In the single session protocol cell death increased with increasing MF incubation time leading to more than 75% cell death after 24 h of MF treatment alone, indicating severe cytotoxicity of magnetic fluid even without induction heating. Subsequently, a multiple session hyperthermia protocol was designed where two consequent 3 h MF incubation followed by 30 min hyperthermia after a gap of 3 h was performed that resulted in more than 90% killing of MCF7 cells. Thus, multiple session hyperthermia was effective in killing a larger cell population compared to the single session protocol. This result suggests that intermittent induction heating followed by shorter MF incubation periods is more effective than hyperthermia conducted after a long MF incubation time. Overall, the study results may help in optimizing protocols for the profused killing of cancer cells of multiple origins besides breast cancer, and also aid in deciding futuristic in vivo MFH-based therapeutic strategies using temperature-sensitive magnetic fluid against breast cancer.
